# Identification of biomarkers and immune infiltration characterization of lipid metabolism-associated genes in osteoarthritis based on machine learning algorithms

**DOI:** 10.18632/aging.205740

**Published:** 2024-04-17

**Authors:** Yuanye Ma, Yang Liu, Dan Luo, Zhu Guo, Hongfei Xiang, Bohua Chen, Xiaolin Wu

**Affiliations:** 1 Department of Orthopedics, The Affiliated Hospital of Qingdao University, Qingdao University, Qingdao 266003, China; 2 Department of Pathology, The Affiliated Hospital of Xuzhou Medical University, Xuzhou 221000, China; 3 Cancer Institute, Qingdao University, Qingdao 266071, China

**Keywords:** osteoarthritis, lipid metabolism associated gene, machine learning algorithms, immune infiltration, biomarkers

## Abstract

Osteoarthritis (OA) is a prevalent degenerative condition commonly observed in the elderly, leading to consequential disability. Despite notable advancements made in clinical strategies for OA, its pathogenesis remains uncertain. The intricate association between OA and metabolic processes has yet to receive comprehensive exploration. In our investigation, we leveraged public databases and applied machine learning algorithms, including WGCNA, LASSO, RF, immune infiltration analysis, and pathway enrichment analysis, to scrutinize the role of lipid metabolism-associated genes (LAGs) in the OA. Our findings identified three distinct biomarkers, and evaluated their expression to assess their diagnostic value in the OA patients. The exploration of immune infiltration in these patients revealed an intricate relationship between immune cells and the identified biomarkers. In addition, *in vitro* experiments, including qRT-PCR, Western blot, chondrocyte lipid droplets detection and mitochondrial fatty acid oxidation measurement, further verified abnormal expressions of selected LAGs in OA cartilage and confirmed the correlation between lipid metabolism and OA.

## INTRODUCTION

Osteoarthritis (OA) is a common senile degenerative disease that causes disability in the elderly [1]. Clinically, OA refers to a disease of the whole joint involving structural alterations in the articular cartilage, subchondral bone, ligaments, capsule, synovial membrane and periarticular muscles [2]. As the world ages and obesity increases, currently, more than 250 million people worldwide are affected by joint injuries, placing a burden on health systems [3, 4]. Although clinical strategies of OA have improved greatly, the pathogenesis of OA remains unclear, joint replacement surgery remains the first choice for OA patients [5]. Previous opinions revealed that mechanical injury, inflammatory, innate immune deficiency and abnormal metabolism factors are involved in the pathogenesis process of OA, causing structural destruction of synovial joint [6–8]. Age is the most important risk factor for OA patients. Other risk factors include heavy work activities, obesity, joint injury and crystal deposition [9, 10]. Additionally, some available clinical evidence indicates an association between OA and abnormal metabolic diseases, such as cardiovascular diseases, diabetes and hypertension [11–13]. A system review showed that over half of the aged population with OA had hypertension, cardiovascular diseases, dyslipidemia and diabetes [14]. Those complications may exacerbate the procession of OA. However, the relationship between OA and metabolic processes has not been thoroughly studied.

There is evidence illustrating the clinical phenotype of abnormal metabolic syndrome-associated OA [15]. Emerging evidence also indicated that low-grade inflammation and lipid metabolism mediate the procession of OA and abnormal metabolic disease [16]. Abnormal lipid metabolism is associated with various diseases, such as NAFLD, diabetes, hypertension and some kinds of malignancies [17–20]. In an earlier study, researcher has proposed a role for lipid in OA. Patients with OA had a significantly higher level of fatty acids and arachidonic acid [8]. Another research reported the relationship between chondrocyte lipid peroxidation and oxidative degradation of OA cartilage matrix proteins [21]. Although many reports suggest alterations in lipid metabolism are involved in the pathogenesis of OA, however, the specific mechanism still needs further study.

It has been reported that infiltration of immune cells is a key factor in promoting the development of OA [22]. The significant infiltration of a variety of immune cells, including neutrophils and macrophages, in OA synovial tissue suggests its ability as a key characteristic marker of OA [23, 24]. However, the research on OA immune infiltration is not sufficient. Although there are clues to the possibility of involvement of the autophagy process [25], further research is needed on the immune infiltrating state of OA.

Currently, the identification of disease feature biomarker based on bioinformatics and genome sequencing technologies has attracted increasing attention [26, 27]. In the present study, we explore the role of lipid metabolism-related genes in the procession of OA using multiple bioinformatics algorithms. Three feature biomarkers were identified, and the diagnostic value of OA patients based on the expression of biomarkers was evaluated. Moreover, we elucidated potential signaling pathways related to the procession of OA. *In vitro* experiments were also performed to further verify abnormal expressions of selected LAGs in OA cartilage and to confirm the correlation between lipid metabolism and OA. This study provides new perspectives for the association between lipid metabolism and OA.

## MATERIALS AND METHODS

### Dataset download

Three microarray datasets comprising normal and osteoarthritis samples, namely GSE51588, GSE98918, and GSE117999, were obtained from GEO datasets. Notably, GSE117999 and GSE98918 utilized the Agilent-072363 SurePrint G3 Human GE v3 8x60K Microarray 039494 platform, while GSE51588 was based on GPL13497 Agilent-026652 Whole Human Genome Microarray 4x44K v2. A total of 34 normal samples and 64 osteoarthritis samples were extracted from GEO datasets, with the breakdown as follows: GSE51588 (10 normal samples, 40 osteoarthritis samples), GSE98918 (12 normal samples, 12 osteoarthritis samples), and GSE117999 (12 normal samples, 12 osteoarthritis samples). All dataset probes were converted into corresponding gene symbols by using Perl scripts and the probe annotation files associated with the datasets. Subsequently, normalization of the matrices from the three distinct GEO datasets and the removal of batch effects were executed through the implementation of “sva” and “limma” scripts.

### Lipid metabolism-associated genes acquisition and difference analysis

Lipid metabolism-associated genes (LAGs) were compiled from the Reactome databases (https://reactome.org/), a total of 1024 LAGs were identified to subsequent analysis (Supplementary Table 1) [28]. To investigate the differential expression of LAGs (DE-LAGs) between the Healthy Control (HC) and OA groups, the “limma” script was employed. The criteria for the identification of DE-LAGs were set at *p*.adjust < 0.05 and |fold change| ≥ 2.

### WGCNA and machine learning model establishment

The “WGCNA” script was employed to establish a weighted gene co-expression network analysis (WGCNA). Initially, the HC and OA samples were clustered to identify and eliminate outlier samples subsequently. Then, the remaining samples were included in subsequent analyses. Using a soft power parameter (β), a WGCNA network was constructed, and the association between clinical features and gene modules was explored. A significant gene module was selected based on correlation coefficients and *p*-values. To investigate diagnostic feature biomarkers, two distinct algorithms were applied utilizing the set of DE-LAGs. The Least Absolute Shrinkage and Selection Operator (LASSO) was conducted to identify the feature variables. Then, random forest (RF) algorithm was performed to calculate the importance of each variable. Support Vector Machine Recursive Feature Elimination (SVM-RFE) algorithms were subsequently employed to identify characteristic genes. The diagnostic feature biomarkers were determined by identifying overlapping genes from the LASSO, RF and SVM-RFE results.

### Functional enrichment analysis

To elucidate potential functional components and pathways, gene ontology (GO) and Kyoto Encyclopedia of Genes and Genomes (KEGG) pathway enrichment analyses were meticulously conducted employing the “clusterProfiler” R packages. The outcomes of these analyses were visually presented through a bubble plot, wherein a *p*.adjust value < 0.05 served as the criterion denoting statistical significance. Furthermore, the Gene Set Enrichment Analysis (GSEA) methodology was employed to enrich the pool of differentially expressed genes within the context of KEGG signaling pathways.

### Analysis of immune infiltration

The assessment of immune cell infiltration levels was accomplished through Single Sample Gene Set Enrichment Analysis (ssGSEA) employing the “GSVA” R package. The intricate relationships among immune cell components were delineated using the “Corrplot” R package. Utilizing the “ggplot2” R package, distinctions in immune cell composition between HC and OA samples were meticulously ascertained. In addition, Spearman correlation analysis was employed to scrutinize the correlation between the three identified characteristic genes and the abundance of immune infiltrating cells. A *p*.adjust value < 0.05 was established to signify statistical significance in these correlation analyses.

### Validation of biomarkers and diagnostic effectiveness analysis

To assess the predictive capability and accuracy of the identified biomarkers, the expression profiles of the three feature genes were scrutinized in a training cohort comprising 34 HC samples and 64 OA samples. The Receiver Operating Characteristic Curve (ROC) was employed to thoroughly investigate the diagnostic effectiveness of the identified biomarkers within the training cohort. A nomogram was intricately constructed based on the feature variates, utilizing the “nomogram” tool to evaluate the diagnostic capabilities. The nomogram provided a quantitative and individualized assessment of the diagnostic potential of the identified genes.

### Real-time quantitative RT-PCR (qRT-PCR) analysis

The OA and HC specimens utilized in this study were acquired with the explicit approval of the human ethics committee at the Affiliated Hospital of Qingdao University and the Ethics Office of Qingdao University, ensuring adherence to ethical standards. RNA extraction from both normal and OA tissues was accomplished using Trizol reagent (Cat# 15596018, Thermo Fisher Scientific, USA), and subsequent cDNA synthesis was conducted utilizing the RT kit with gDNA Eraser (Perfect Real Time). Real-time quantitative reverse transcription polymerase chain reaction (qRT-PCR) (Cat# RR047A, Takara, Japan) was employed for further analysis. The mRNA expression levels were discerned using SYBR Pre-mix Ex Taq II (TliRNaseH Plus) (Cat# RR820B, Takara). The assessment of relative RNA expression levels was conducted employing the 2^−ΔΔCT^ method. The primer sequences utilized in this analysis are elucidated in Supplementary Table 2.

### Western blot analysis

Total protein extraction from OA and HC samples was achieved using RIPA lysis buffer (Cat# R0010, Solarbio, China). Subsequently, the quantification of total protein was performed utilizing the BCA assay kit (Cat# PC0020, Solarbio). A 20 μL protein sample was combined with 200 μL of BCA working solution and incubated at 37°C for 30 minutes. The absorbance at 562 nm was measured using a spectrophotometer (CMax Plus, USA), and the protein concentration was determined through reference to the standard curve of BSA. Following quantification, protein samples were mixed with loading buffer at a ratio of 4:1 (v/v), boiled for 10 minutes, subjected to sodium dodecyl sulfate-polyacrylamide gel electrophoresis (SDS-PAGE), and subsequently transferred to a polyvinylidene fluoride (PVDF) membrane. The PVDF membrane underwent blocking with 5% skimmed milk powder for 1 hour at room temperature. Following this, the membrane was incubated overnight at 4°C with primary antibodies targeting β-actin (Cat# E-AB-40517, Elabscience, USA), JUN (Cat# ab31367, Abcam, UK), LTC4S (Cat# PA5-49613, Abcam), and NFKBIA (Cat# 10268-1-AP, Proteintech, China). After washing with TBST buffer solution three times, the transferred membrane was incubated with the secondary antibody (1:20000) at room temperature for 1 hour, followed by additional washes with TBST. Ultimately, the protein bands were visualized using an Odyssey Clx system (Li-Cor, USA). Blots were imaged and quantified utilizing ImageJ software, with β-actin serving as a loading control.

### Cartilage cell isolation and culture

OA and normal cartilage tissues were obtained as discarded specimens from the hospital, and divided into superficial and middle layers within a 2-hour timeframe expeditiously. Subsequently, the collected cartilage was subjected to enzymatic digestion using 0.2% type II collagenase (Cat# C8150, Solarbio) in DMEM (Cat# 31600034, Solarbio) at 37°C for 3 hours. The resulting cells were then filtered through a 70-μm nylon cell strainer and harvested via centrifugation at 250 g for 5 minutes. The cells were resuspended in DMEM culture medium enriched with 10% FBS (Gibco, USA) and 1% penicillin/streptomycin (Gibco, USA). These cells were then seeded in 60 mm diameter culture dishes, with the culture medium being refreshed every 3 days to maintain optimal conditions for cell growth and viability.

### Detection of lipid droplets in chondrocytes

For the visualization of lipid droplets (LD), chondrocytes were cultured in DMEM at 37°C with the addition of 10 μM BODIPY 493/503 (Cat# HY-W090090, MedChemExpress, USA) for a duration of 30 minutes. Following this incubation period, the cells underwent three washes before imaging. The fluorescence emanating from BODIPY 493/503 was excited at 488 nm, and the emitted fluorescence was collected within the range of 500–550 nm. This method facilitated the specific and efficient visualization of lipid droplets within the chondrocytes, providing valuable insights into lipid metabolism and distribution in the cellular context.

### Measurement of mitochondrial fatty acid oxidation

Isolated chondrocytes were meticulously seeded at a density of 300 cells per well within XF24 Cell Culture Microplates. The culture medium was subsequently replaced with PBS buffer, and chondrocytes underwent an incubation period at 37°C for 1 hour before commencing measurements. In preparation for the XF BSA-Palmitate FAO assay, a 1 mM BSA-Palmitate solution and a 0.17 mM BSA solution were precisely prepared following the guidelines provided in the XF BSA-Palmitate FAO assay kit (Cat# 102720-100, Agilent, USA). At specific time points, BSA-Palmitate ester or BSA was introduced through injection. The quantification of mitochondrial fatty acid oxidation was derived by subtracting the Oxygen Consumption Rate (OCR) in the presence of BSA-Palmitate ester from the OCR observed in the presence of BSA. This assay offers a comprehensive assessment of cellular metabolic activity, specifically focusing on mitochondrial fatty acid oxidation dynamics in chondrocytes.

### Statistical analysis

The statistical analyses were conducted using R (version 4.1.0) and Perl software. The disparity between two groups was assessed utilizing the Wilcoxon Test, with a *p*.adjust value < 0.05 deemed as the threshold denoting statistical significance.

## RESULTS

### Characteristics gene screening by WGCNA analysis

**Figure 1:**
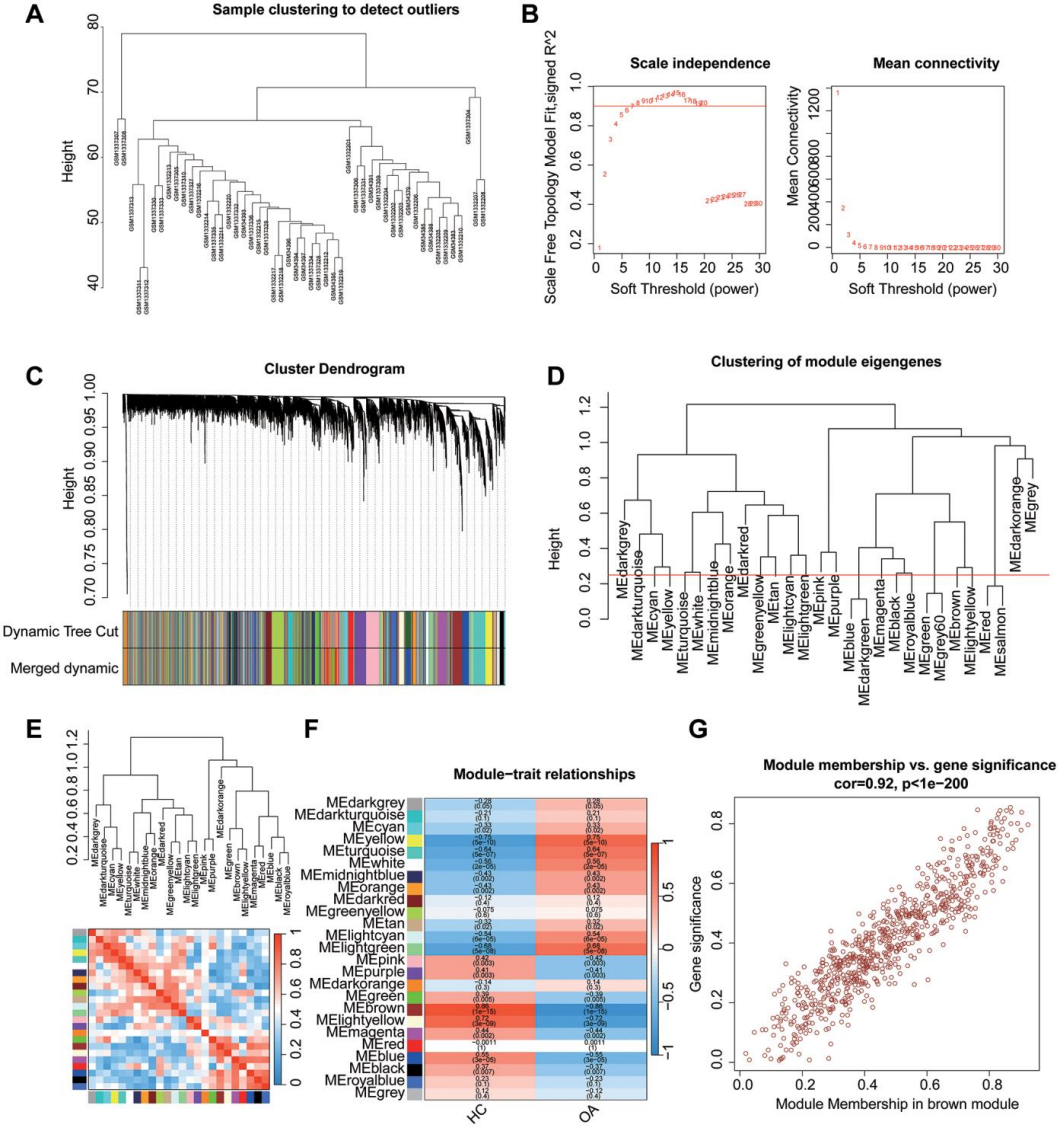
**WGCNA analysis to select characteristics gene module for OA.** (**A**) Clustering of mod. (**B**) Scale free topology model fit and mean connectivity. (**B**) Clustering of module genes. (**C**) Cluster dendrogram for selecting gene modules. (**D**) Clustering of module genes. (**E**) Association between the gene modules. (**F**) Heatmap analysis of 18 modules and clinical features (HC, OA). (**G**) Module membership vs. gene significance in brown module.

We gathered a total of 34 HC and 64 OA samples from three GEO datasets (GSE117999, GSE98918, and GSE51588). After performing sample clustering, all data underwent normalization and were consolidated into a matrix for subsequent analysis (Figure 1A). To identify potential regulatory genes associated with OA, we conducted WGCNA based on the expression of differentially expressed lipid-associated genes, resulting in the construction of a gene co-expression network. A soft thresholding power (β) of 7 was selected to achieve a scale-free network (Figure 1B) with a scale-free R^2^ greater than 0.85. The cluster dendrogram depicted the height of each module, which was further refined using dynamic tree cutting to yield distinct modules (Figure 1C). By setting the clustering height of module eigengenes at 0.25, 25 gene modules were obtained for subsequent analysis (Figure 1D). The correlation heatmap indicated no discernible correlation between each module (Figure 1E). The association between gene modules and clinical features revealed that the light green module was negatively correlated with HC (r = −0.68, *p* = 5e-08), while positively correlated with OA (r = 0.68, *p* = 5e-08). Conversely, the brown module showed a positive correlation with HC (r = 0.86, *p* = 1e-15) and a negative correlation with OA (r = −0.86, *p* = 1e-15). Similarly, the light-yellow module displayed a positive correlation with HC (r = 0.72, *p* = 3e-09) and a negative correlation with OA (r = −0.72, *p* = 3e-09, Figure 1F). Given the highest correlation coefficient, the brown module was identified as the most characteristic module. The subsequent scatter plot demonstrated a high correlation (r = 0.92, *p* < 1e-200) between module brown membership and gene significance. The genes within the brown module were selected for further analysis (Figure 1G).

### Identification of diagnostic feature biomarkers

**Figure 2:**
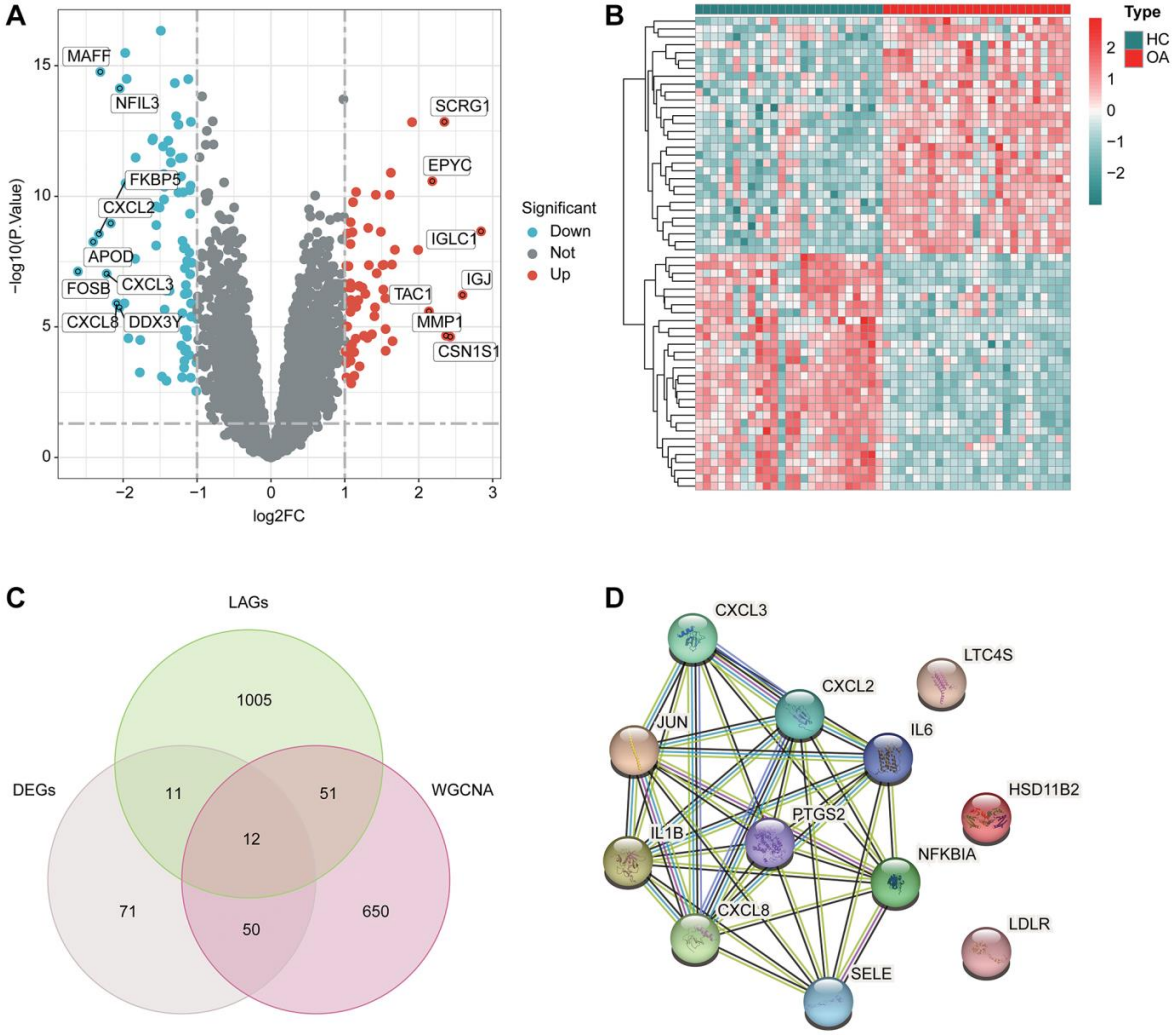
**DE-LAGs screening.** (**A**) Volcano plot of DEGs in HC and OA groups. The threshold of screening DEGs is set at |fold change| ≥ 2 and *p*.adjust < 0.05. Turquoise dots represent down-regulated genes and red dots represents up-regulated genes. (**B**) Analysis of top 25 up- and down-regulated genes in HC and OA group. (**C**) Identification of pivotal DE-LAGs in brown module. (**D**) Protein-protein interaction (PPI) network analysis among screened genes.

In the pursuit of identifying DE-LAGs between HC and OA samples, we applied stringent screening conditions, setting |fold change| ≥ 2 and a *p*.adjust value < 0.05 threshold. This analysis resulted in the identification of a total of 291 DE-LAGs, comprising 90 genes that were significantly up-regulated and 201 genes that were significantly down-regulated (Figure 2A). The heatmap diagram vividly displayed the expression profiles of the top 25 regulated DEGs in both directions for HC and OA (Figure 2B). Through an integrated analysis involving the WGCNA specifically focusing on the brown module, and the aforementioned DE-LAGs, we identified 12 pivotal genes at the intersection by employing a Venn diagram (Figure 2C). Subsequently, a Protein-Protein Interaction (PPI) network analysis was conducted, revealing potential interactions among these identified genes (Figure 2D). This comprehensive approach enhances our understanding of the key regulatory genes associated with lipid metabolism in the context of OA.

### Functional enrichment analysis of pivotal module genes

**Figure 3:**
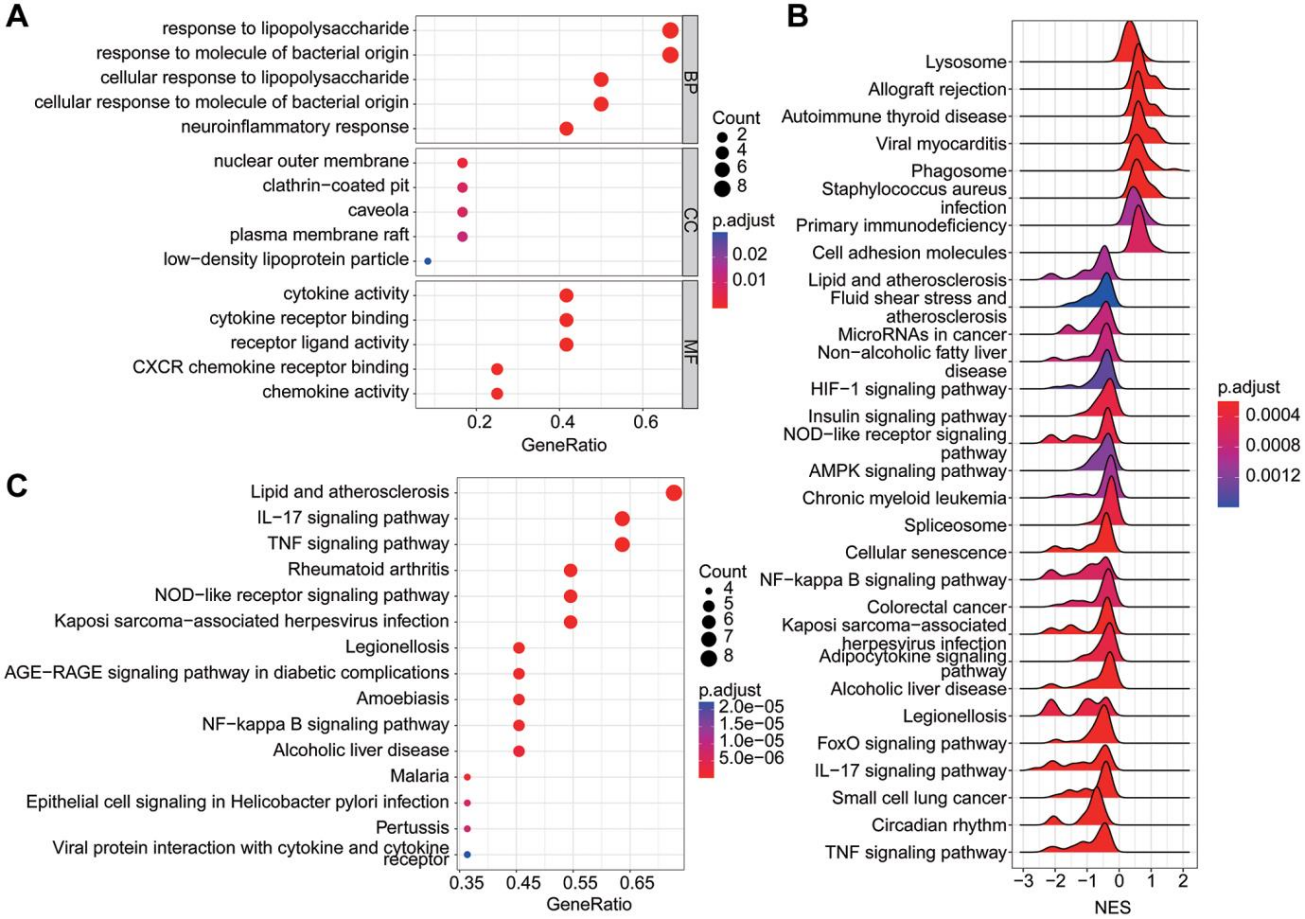
**Function enrichment analysis of DE-LAGs.** (**A**) Gene ontology (GO) analysis of DE-LAGs in HC and OA. (**B**) GSEA analysis of DEGs in HC and OA group. (**C**) Kyoto Encyclopedia of Genes and Genomes (KEGG) analysis of DE-LAGs in HC and OA.

We employed functional enrichment analysis to delve into the potential molecular biological functions of the pivotal DE-LAGs in the context of OA. The Gene Ontology (GO) enrichment analysis unveiled that these pivotal DE-LAGs were notably associated with the response to lipopolysaccharide and the response to molecules of bacterial origin (Figure 3A). Further exploration through Gene Set Enrichment Analysis (GSEA) revealed that the differentially expressed genes in the OA group exhibited significant enrichment in pathways such as lysosome, allograft rejection, and autoimmune thyroid disease. Conversely, DEGs in the HC group were prominently enriched in immune-related signaling pathways, including lipid and atherosclerosis, fluid shear stress and atherosclerosis, microRNAs in cancer, and non-alcoholic fatty liver disease (Figure 3B). The Kyoto Encyclopedia of Genes and Genomes (KEGG) analysis of the pivotal DE-LAGs demonstrated associations with lipid and atherosclerosis, as well as the IL-17 and TNF signaling pathways (Figure 3C). This comprehensive analysis sheds light on the diverse molecular functions and pathways implicated by the identified DE-LAGs, providing valuable insights into the intricate mechanisms underlying OA.

### Feature biomarkers selection via machine learning algorithms

**Figure 4:**
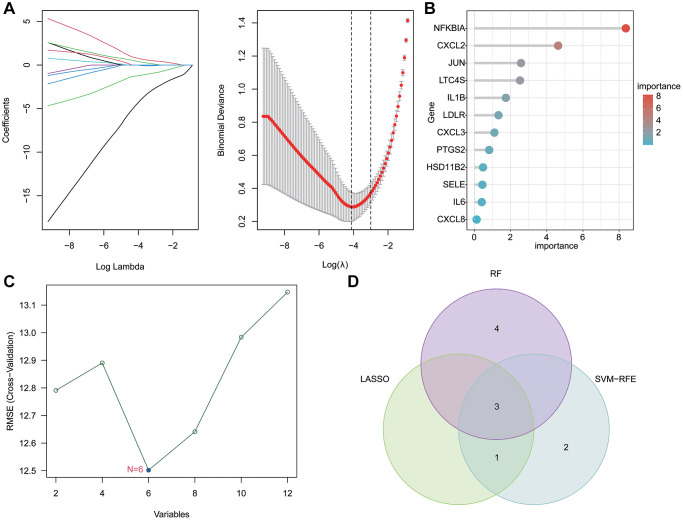
**Feature biomarkers selection via machine language algorithms.** (**A**) Key LAGs screening by LASSO analysis. (**B**) RF analysis of key DE-LAGs, the filter condition for screening feature variates was set at: importance > 3. (**C**) SVM-RFE algorithm for selecting the feature DE-LAGs. (**D**) Venn network plot showed the three diagnostic feature biomarkers based on LASSO, SVM-RF and RF algorithm.

We proceeded with the application of several machine learning algorithms to discern the feature DE-LAGs associated with OA. LASSO algorithm revealed the minimum lambda of DE-LAGs, identifying 4 characteristic variates (Figure 4A). Simultaneously, the RF algorithm yielded 7 feature DE-LAGs for subsequent analysis (Figure 4B). The SVM-RFE algorithm results indicated 6 DE-LAGs as feature variables (Figure 4C). Upon integration of results from SVM-RFE, LASSO and RF algorithms, three DE-LAGs (NFKBIA, LTC4S, and JUN) were ultimately determined as the feature variables (Figure 4D).

### Evaluation of the diagnostic validity of biomarkers for LAGs

**Figure 5:**
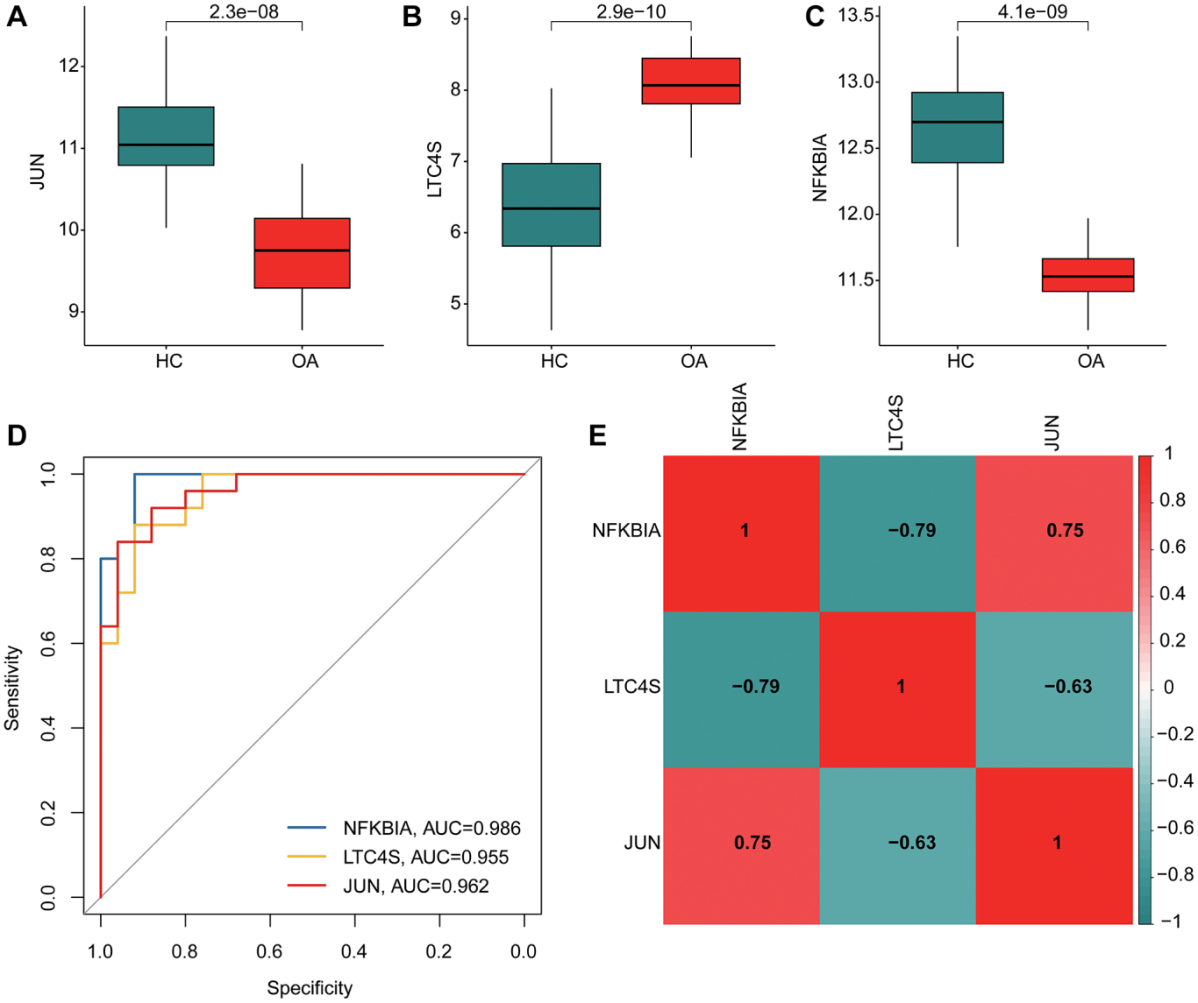
**Immune cell infiltration analysis of HC and OA based on CIBERSORT algorithm.** (**A**–**C**) The expression of JUN, NFKB1A and LTC4S in OA and HC groups. (**D**) Nomogram construction and ROC curve of three gene signatures. (**E**) Correlation heatmap of BCKDHB, LETMD1, and NDUFB3. Green color represents negative correlation, red color represents positive correlation.

To validate the expression levels and diagnostic efficacy of the feature biomarkers, the expressions of the three selected biomarkers were analyzed. The HC group exhibited higher expression levels of JUN and NFKB1A, and lower expression of LTC4S (Figure 5A–5C). Furthermore, a nomogram model was meticulously constructed to assess the diagnostic efficacy based on the three-gene signatures. The results of the nomogram illustrated a satisfactory diagnostic ability of JUN, NFKB1A, and LTC4S for OA (Figure 5D). Additionally, as depicted in Figure 5E, a significant association was observed among the three feature biomarkers. This comprehensive analysis validates both the differential expression patterns and the diagnostic potential of the selected biomarkers in the context of OA.

### Immune infiltration landscape analysis

**Figure 6:**
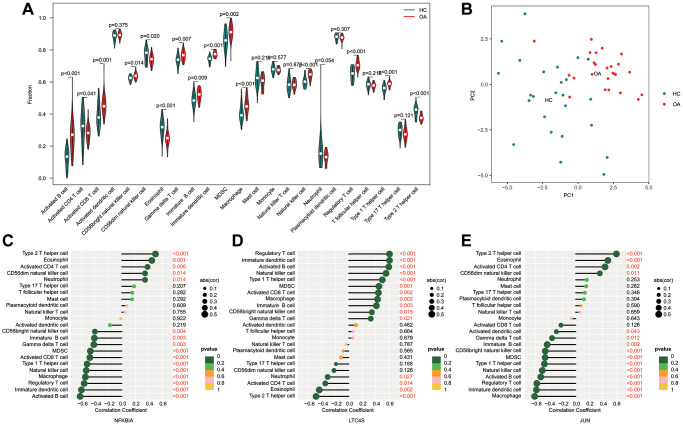
**Immune cell infiltration analysis of HC and OA based on ssGSEA algorithm.** (**A**) Immune infiltration analysis of 23 type immune cells by ssGSEA. (**B**) Principal components analysis (PCA) between HC and OA groups. (**C**–**E**) Correlation analysis of three diagnostic biomarkers (JUN, NFKB1A and LTC4S) and immune microenvironment.

A prior study has highlighted the association of OA with the immune system. Consequently, we explored the composition of 23 immune cells utilizing the ssGSEA algorithm. The ssGSEA results indicated that OA exhibited significantly higher fractions of activated B cells, activated CD8 T cells, γδT cells, immature B cells, immature dendritic cells, Myeloid-Derived Suppressor Cells (MDSCs), macrophages, Natural Killer (NK) cells, regulatory T cells, and type 1 T helper cells. Conversely, the HC group demonstrated higher fractions of activated CD4 T cells, eosinophils, and type 2 T helper cells (Figure 6A). A Principal Components Analysis (PCA) plot further illustrated a distinct distribution pattern between the HC and OA groups (Figure 6B). Additionally, correlation analysis was conducted to examine the individual effects of the three screened diagnostic biomarkers (JUN, NFKB1A, and LTC4S) on immune infiltration (Figure 6C–6E). This comprehensive analysis provides valuable insights into the differential immune cell composition and distribution patterns between HC and OA, as well as the potential correlation between the diagnostic biomarkers and the immune microenvironment.

### qRT-PCR and Western blot validation in clinical samples

**Figure 7:**
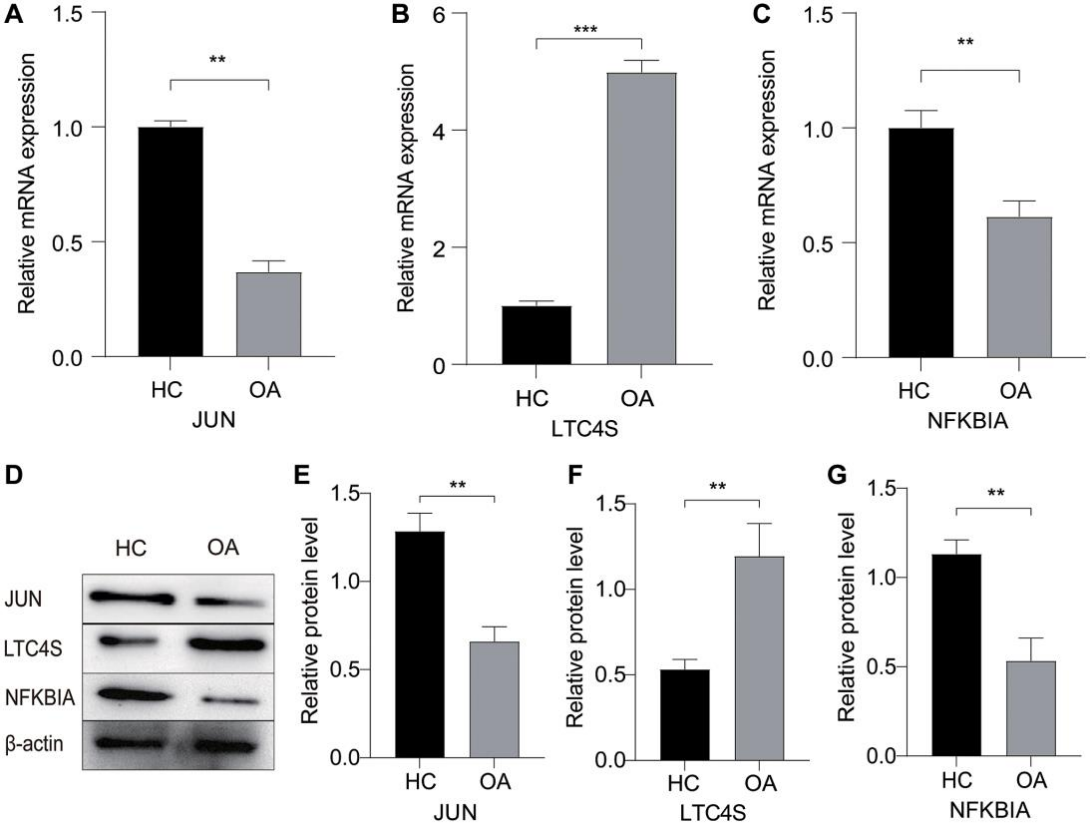
**The expression profile of relevant genes and proteins in OA.** (**A**–**C**) Changes in mRNA expression levels of JUN, LTC4S, and NFKBIA in the OA and HC groups. (**D**–**G**) Western blotting (WB) analysis of the protein expression levels of JUN, LTC4S, and NFKBIA in OA and HC. ^*^p < 0.05, ^**^p < 0.01, ^***^p < 0.001.

In our further exploration of the mRNA and protein expressions of selected biomarkers using clinical samples, the quantitative real-time PCR (qRT-PCR) results (Figure 7A–7C) elucidated that the mRNA expressions of JUN and NFKB1A were distinctly lower in OA patients compared to healthy donors. Conversely, the mRNA expression of LTC4S was markedly higher in OA patients than in the normal control group. Subsequent Western blot analysis (Figure 7D–7G) further affirmed that the trends of these three biomarkers were consistent at the protein level. These findings provide a partial validation of our bioinformatics results, reinforcing the evidence of altered expression patterns of the identified biomarkers in the context of OA when assessed at both the mRNA and protein levels.

### Impaired fatty acid oxidation in OA leads to the accumulation of lipid droplets within the cells

**Figure 8:**
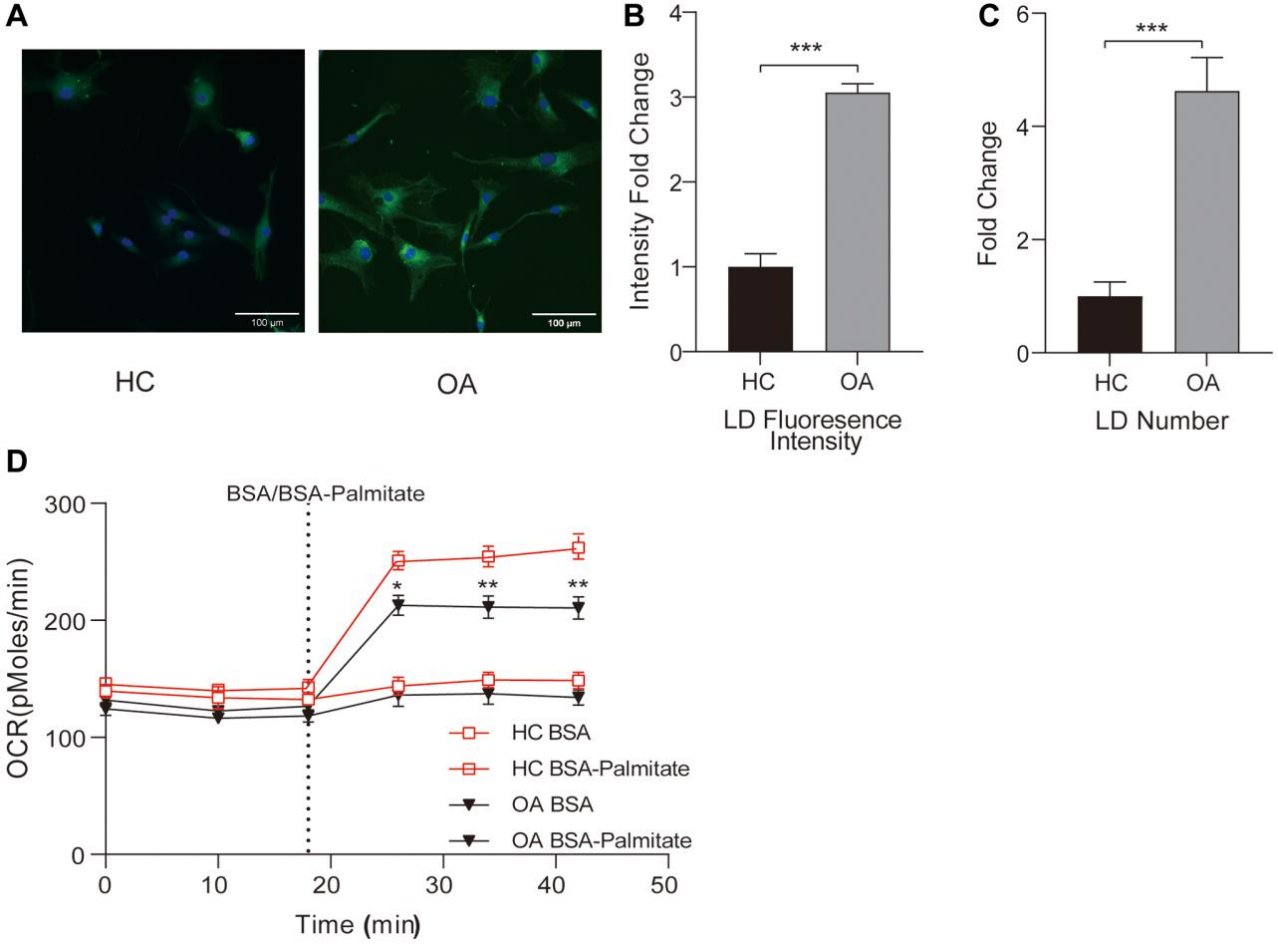
**The changes in intracellular lipid metabolism in OA.** (**A**) Representative images of intracellular lipid droplets stained with BODIPY 493/503. The left side shows normal chondrocytes, and the right side shows OA chondrocytes; Scale bar: 100 μm. (**B**) Multiplicative changes in the average fluorescence intensity of intracellular lipid droplets stained with BODIPY 493/503. (**C**) Multiplicative changes in the number of lipid droplets stained with BODIPY 493/503. (**D**) Measurement of changes in fatty acid oxidation (FAO) in cells from the Normal and OA groups. Dashed lines represent the time points of adding BSA (0.17 mM) or BSA-Palmitate ester (1 mM). ^*^p < 0.05, ^**^p < 0.01, ^***^p < 0.001.

In utilizing isolated chondrocytes to investigate alterations in fatty acid metabolism, the visual assessment of lipid droplets (LD) reveals a conspicuous increase in LD accumulation within isolated OA chondrocytes as compared to the negative control (HC) group. This is evident in both a higher average fluorescence intensity and a greater quantity of LD in each OA chondrocyte (Figure 8A–8C). Furthermore, the Fatty Acid Oxidation (FAO) in the BSA-Palmitate ester group within the OA group is significantly lower than in the HC group (Figure 8D). These results collectively indicate a marked inhibition of fatty acid oxidation (FAO) in OA chondrocytes, signifying disrupted lipid metabolism within the cartilage tissue of OA patients. This disruption contributes to the accumulation of lipid droplets within the chondrocytes, providing valuable insights into the aberrations in lipid metabolism associated with OA.

## DISCUSSION

OA is one of the main causes of disability in the elderly and has become a source of social burden. Prevention and disease modification has suggested great potential in the treatment of OA. Recently, researchers are increasingly finding novel feature biomarkers of disease which provide contribution to the clinical benefit. For example, ADAMTS-5 and IL-1β could predict the prognosis of OA [29]. PRKACB could serve as a biomarker to access the risk and indicate the immune infiltration of OA [30]. However, few studies reported the role of LAGs in OA. Therefore, we aimed to investigate the diagnostic feature biomarkers for OA and explore the association of LAGs and immune infiltration in OA.

We focus on the role of lipid metabolism in OA. Lipid metabolism was considered an important mechanism involved in disease regulation. In the past decades, studies have indicated that abnormal lipid metabolites associated with cancer, non-alcohol fatty liver disease (NAFLD), diabetes and Alzheimer’s disease. Abnormal lipid metabolism is associated with immune microenvironment status [31]. Reversing excessive fat accumulation can effectively reverse NAFLD process [32], indicating targeting lipid metabolism process may be an effective strategy for disease treatment.

Metabolic disorders such as obesity and diabetes have been identified as risk factors for OA. Evidence further suggests that lipid metabolism, as a common pathway of metabolic disease and OA, may have direct systemic effects on the joints [8]. There are two modes of lipid transport: synovial diffusion and subchondral bone exchange [33]. As the main source of molecules in articular cartilage metabolism, synovial fluid can provide sufficient nutrients to maintain the structure and function of mature articular cartilage [33]. Lipid transport is facilitated by uncalcified cartilage. However, calcified cartilage is found at the bone-cartilage interface in mature joints. This natural barrier greatly limits the passage of lipids from calcified cartilage to non-calcified cartilage [34]. Therefore, abnormal lipid accumulation in OA chondrocytes is detrimental to cartilage nutrition and contributes to the occurrence and progression of OA [35]. In addition, as the second messenger between cells, lipids play an important role in OA signal transduction, which also indicates that lipids play an important role in the occurrence and development of OA [36]. The rationalization of daily dietary lipids has also been shown to have a slowing effect on the course of OA [37]. Therefore, further investigation of the influence of lipid metabolism would be a promising direction for the treatment of OA in the internal joint disease [38]. To help achieve this goal, we preliminarily explored the OA associated LAGs and identified three diagnostic feature biomarkers. The results showed that three feature biomarkers have a diagnostic value and could indicate the immune microenvironment of OA.

The functional enrichment analysis of de-lag showed that IL-17, TNF, NOD-like receptor and other pathways may be involved in the formation of OA. In OA patients, IL-17 not only affects the inflammatory response, angiogenesis and glycolytic pathways of chondrocytes and synovial fibroblasts, but also is closely related to the degree of joint pain in OA patients [39, 40]. The TNF pathway has also been reported to be associated with inflammatory responses in articular chondrocytes and as a potential therapeutic target [41, 42]. Rheumatoid arthritis (RA), also a disease affecting joints, shares certain proteins with OA and has been shown to affect disease progression [43, 44]. NOD-like receptor related pathways have been poorly studied in OA, but there is also evidence that Nod-like receptor protein-3 can inhibit chondrocyte pyroptosis and alleviate cartilage damage in osteoarthritis [45]. Our results once again confirm the important role of the above signaling pathways in the development of OA.

JUN-related pathway is one of the key signaling pathways in autoimmune diseases and is closely related to physiological processes such as cell proliferation, cell differentiation, cell survival, cell death and immune response [46]. In OA, continuous inflammatory stimulation can cause the constitutive expression of JUN, and may act as a signal transmitter to further activate the inflammatory response, thus forming the intrinsic activation mechanism of OA [47]. Multiple pathways have been shown to be related to JUN’s promoting effect on OA [48–50], and inhibition of JUN transcription can prevent osteoarthritic cartilage destruction [51, 52]. Our results further confirm the potential role of JUN in OA and its potential application as an intervention target.

LTC4S is believed to be a mediator in the development of allergic reactions and inflammatory diseases such as bronchial asthma [53]. It has been reported that LTC4S expression is up-regulated in OA synovium [54]. However, beyond that, we could not find more evidence for its role in OA development. As another key gene we screened, NFKBIA, has not been studied in OA. In the only study that showed a systematic review based on a Han population, NFKBIA was significantly associated with hip OA [55]. NFKBIA encodes IκBα, which binds to NF-κB proteins p65 and p50, and acts as one of the inhibitors of NF-κB activation [56]. The activated NF-κB signaling pathway can induce joint destruction, leading to the occurrence and development of OA [57]. In addition, NFKBIA has been reported to play an integral role in macrophage-mediated inflammatory responses [58]. Combined with the important role of macrophages in OA development and their great potential as therapeutic targets [59], the value of NFKBIA in OA development and therapeutic intervention deserves further attention.

Lipid metabolism has shown its critical role in bone metabolism [38]. In this study, the comparison of normal and OA samples showed that DE-LAGs were involved in a variety of lipid biological functions and lipase activity, glycolide metabolism, phospholipid metabolism, and lipid and atherosclerosis signaling pathways. The activity of lipase also suggested the role of lipid metabolism in OA. Although limited by conditions, no *in vivo* experiment was conducted, this study provides a new idea for further experimental verification and a few potential targets for OA risk stratification.

## Supplementary Materials

Supplementary Table 1

Supplementary Table 2
