# Supplementary material for: Identification of biomarkers and immune infiltration characterization of lipid metabolism-associated genes in osteoarthritis based on machine learning algorithms
Source: Aging (Albany NY). 2024 Apr 17;16(8):7043–59. doi: 10.18632/aging.205740 (PMC11087088; doi:10.18632/aging.205740)
Supplement: Supplementary Table 2 [file aging-16-205740-s002.pdf]

SUPPLEMENTARY TABLE

Supplementary Table 2. The primers used for qPCR detection.

Gene name	Primer sequences (5′–3′)
GAPDH	Forward (F) 5′-ATGCCGCCTGGAGAAACC-3′<br>Reverse (R) 5′-GCATCAAAGGTGGAAGAATGG-3′
JUN	Forward (F) 5′-CAGCCAGGTCGGCAGTATAG-3′<br>Reverse (R) 5′-GGACTCTGCCACTTGTCTCC-3′
LTC4S	Forward (F) 5′-CCGACGGTACCATGAAGGAC-3′<br>Reverse (R) 5′-GCAGGGAGAAGTAGGCTTGC-3′
NFKBIA	Forward (F) 5′-ATGTCAATGCTCAGGAGCCC-3′<br>Reverse (R) 5′-GGTCAGTCACTCGAAGCACA-3′
